# Retrospective gating leads to more accurate velocity measurements than prospective gating in spiral phase velocity mapping

**DOI:** 10.1186/1532-429X-16-S1-P383

**Published:** 2014-01-16

**Authors:** Robin Simpson, Jennifer Keegan, David Firmin

**Affiliations:** 1 Royal Brompton Hospital, Imperial College, London, UK

## Background

Spiral phase velocity mapping (PVM) has been used to assess both blood flow [1] and myocardial velocities [2]. Data is usually acquired over several heartbeats which, will not be exactly the same due to physiological RR interval (RR) variations. The way in which the data from different cycles is combined depends on the method of ECG gating. This simulation study investigates the effect of RR variation on the velocities measured for prospective (pro) and retrospective (retro) gating.

## Methods

**Figure 1:**
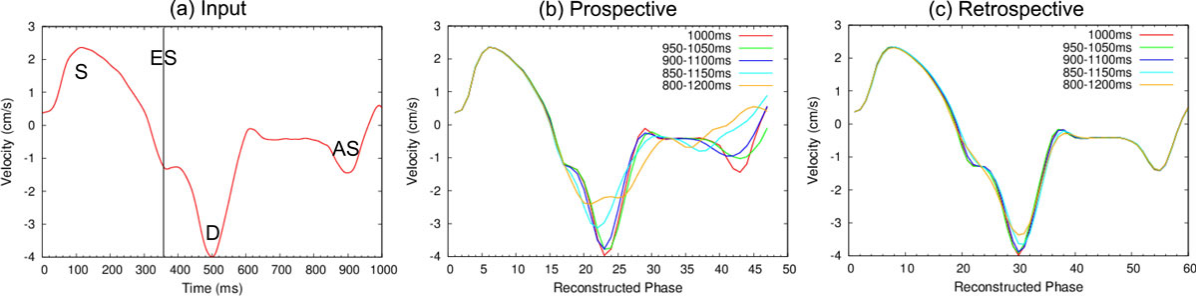
**a) Input curve derived from radial myocardial velocity measurements in healthy volunteers**. Three peaks are seen in systole (S), diastole (D) and atrial systole (AS). End systole (ES) is marked as a vertical line. For a given RR duration the curve is split into systole and diastole and each section is stretched according to the given formula. Curves from each of the 13 RR durations that make up a full acquisition are then combined according to the ECG gating algorithm being simulated. Simulated curves in the presence of RR variation are shown for prospective (b) and retrospective (c) gating.

Each spiral path is the same except for a rotation around the centre of k-space and hence each contributes equally to the final image. The final velocity-time curve reconstructed from a multi-spiral trajectory can therefore be estimated as the average of the velocity-time curves which would be generated by each spiral individually. This simulation study assumes data acquisition over 13 spiral interleaves, with one interleaf acquired per heartbeat. The input velocity-time curve used (Figure 1a) is typical of radial myocardial velocities in a short-axis view [2], with characteristic peaks in systole (S), early diastole (D) and atrial systole (AS). Sets of 13 RRs are randomly generated with 5%, 10%, 15% and 20% variation around a base RR of 1 second. Systole and diastole are then stretched separately (systole = 0.456 - 108/RR, diastole = RR - systole [3]). To simulate pro, the 13 stretched curves are then averaged at each time point. To simulate retro, the 13 curves are normalised to the same duration, sampled at equal time intervals and then averaged at each time point. Errors in S, D and AS peak and time to peak velocity for both retro and pro gating were determined for all levels of RR variation.

## Results

Results are shown in Figure 1 and Table 1. S is well preserved at all levels of RR variation for both pro and retro gating techniques. D is progressively underestimated as RR variation increases from 5% to 20% with the underestimation being greater for prospective gating at all levels of RR variation. AS is severely affected for pro even with low levels of RR variation whereas for retro it is consistently well preserved.

**Table 1:** Percentage errors for peak and time to peak S, D and AS velocities for prospective and retrospective gating

	5% Pro	10% Pro	15% Pro	20% Pro	5% Retro	10% Retro	15% Retro	20% Retro
Peak S	0.1	-0.0	-0.0	0.3	0.0	-0.2	-0.2	0.8

Peak D	4.6	4.9	21.1	39.2	1.7	2.7	8.3	15.5

Peak AS	28.6	34.1	44.3	52.6	0.5	0.6	0.6	1.1

Time S	0.0	0.0	0.0	0.0	-4.6	0.0	0.0	-9.5

Time D	-0.5	-0.5	4.1	8.6	-2.9	0.0	-3.7	-5.7

Time AS	1.9	6.5	15.9	18.2	-0.3	0.0	0.0	-0.5

Error is calculated as percentage difference between measured values with variation and measured value with no variation. Positive error represents underestimation of peak value.

## Conclusions

These simulations show that for this application retro allows more accurate measurements of peak velocities in the presence of heart rate variation than pro: at 15% RR variation, D and AS are both underestimated by more than 20% with pro but by less than 10% with retro. This is because during the normalisation step retro effectively stretches each heartbeat linearly before combining to produce an image while pro makes no attempt to deal with variation. Pro therefore averages velocities from different parts of the cardiac cycle in the presence of RR variation, while the linear stretching in retro goes some way towards reducing the effect. These results show that incorporation of more physiological stretching algorithms could further improve future retro algorithms.

## Funding

CBRU Royal Brompton Hospital. HRUK Grant number RG2584.
